# A pilot study of high-intensity interval training and continuous positive airway pressure on vascular health in Thai adults with obesity, obstructive sleep apnea, and type 2 diabetes

**DOI:** 10.1016/j.pmedr.2025.103238

**Published:** 2025-09-11

**Authors:** Kanpiraya Nithitsutthibuta, Sureeporn Uthaikhup, Nuntigar Sonsuwan, Jaruta Kunritt, Khajornsak Tragoolpua, Arisa Parameyong, Pongson Yaicharoen, Sainatee Pratanaphon

**Affiliations:** a Department of Physical Therapy, Faculty of Associated Medical Sciences, Chiang Mai University, Chiang Mai, Thailand; b Department of Otolaryngology, Faculty of Medicine, Chiang Mai University, Chiang Mai, Thailand; c Department of Medical Technology, Faculty of Associated Medical Sciences, Chiang Mai University, Chiang Mai, Thailand; d Department of Physiology, Faculty of Medicine, Chiang Mai University, Chiang Mai, Thailand

**Keywords:** Carotid intima-media thickness, Flow-mediated dilatation, High-intensity interval training, Continuous positive airway pressure, Obstructive sleep apnea, Type 2 diabetes mellitus

## Abstract

**Objective:**

This pilot study compared the effects of high-intensity interval training (HIIT) and continuous positive airway pressure (CPAP) on vascular health in Thai adults with obesity, obstructive sleep apnea (OSA), and type 2 diabetes mellitus.

**Methods:**

Participants were recruited from a university hospital in Chiang Mai, Thailand (July 2023–April 2024). Thirty participants were age-matched and sequentially assigned to CPAP, HIIT, or usual care groups. The HIIT group completed four 4-min treadmill intervals at 85 %–95 % of maximum heart rate, three times weekly for 12 weeks. The CPAP group used CPAP ≥ four hours/night. Primary outcomes were flow-mediated dilation (FMD) and carotid intima-media thickness (CIMT). Secondary outcomes included sleep indices, blood biomarkers, and body composition.

**Results:**

Adherence was high in both HIIT (97.78 %) and CPAP (95.89 %) groups. The HIIT group showed lower CIMT than controls (*p* = 0.05) and improved FMD and CIMT from baseline (*p* < 0.01). Both HIIT and CPAP groups reduced apnea-hypopnea index and body mass index compared with controls and baseline (*p* ≤ 0.01). Only the CPAP group improved oxygen saturation (p ≤ 0.01). No significant changes were observed in blood biomarkers (*p* > 0.05).

**Conclusions:**

HIIT may benefit vascular health and OSA severity. Both HIIT and CPAP improved body mass index. Larger trials are warranted.

## Highlights

• Interval training was feasible in adults with obesity, sleep apnea, and diabetes.
• High-intensity interval training may improve vascular function and structure.
• High-intensity interval and airway pressure therapy may reduce apnea severity.
• Airway pressure therapy may improve oxygen saturation but not vascular health.
• Further research with larger samples is warranted.

## 1 Introduction

Obesity is a major global health concern and a well-established risk factor for cardiometabolic diseases, including type 2 diabetes mellitus (T2DM) and obstructive sleep apnea (OSA). Evidence over the past two decades, including observational and interventional studies, has shown an association between OSA and T2DM. OSA is highly prevalent in individuals with T2DM, with estimates ranging from 15.2 % to 90.3 % depending on diagnostic criteria and population characteristics (Andayeshgar et al., 2022). Similarly, the prevalence of T2DM is higher among individuals with OSA (30.1 %) than those without OSA (18.6 %) (Mahmood et al., 2009). These conditions share a bidirectional pathophysiological relationship in which each can exacerbate the other. OSA-induced intermittent hypoxia and sleep fragmentation contribute to insulin resistance and inflammation, whereas T2DM-related neuropathy may worsen sleep-disordered breathing by impairing ventilatory control and promoting upper airway collapse (Framnes and Arble, 2018). Both OSA and T2DM independently impair endothelial function, and their coexistence may accelerate vascular deterioration, promoting atherosclerosis, increasing cardiovascular risk, and premature mortality in this population (Bakker et al., 2020).

Continuous positive airway pressure (CPAP) is the standard treatment for individuals with OSA. CPAP has been shown to improve glycemic control and reduce glucose fluctuations in patients with OSA and T2DM (Zhao et al., 2022). However, its impact on vascular health remains inconsistent. While some studies report improvements in endothelial function (Kayastha et al., 2021) and reductions in vascular thickness (Amin, 2016), others have found no significant effects on endothelial function (Bakker et al., 2020) or carotid thickness (Ng et al., 2017). Additionally, the effectiveness of CPAP is often compromised by poor patient adherence. Research indicates that nearly half of patients discontinue CPAP use within a year, while 8–15 % reject it on the first night (Contal et al., 2021). Additional barriers include long wait times and affordability issues (Nogueira et al., 2020). These challenges highlight the need for alternative interventions, such as exercise, which has shown positive vascular outcomes on both OSA and T2DM conditions.

High-intensity interval training (HIIT) is a time-efficient exercise method that alternates short periods of intense activity with rest or low-intensity recovery. It has been shown to be safe for vulnerable populations, including individuals with coronary artery disease, heart failure, hypertension, metabolic syndrome, and T2DM (Wewege et al., 2018). HIIT improves vascular function, cardiorespiratory fitness (CRF), glycemic control, and overall metabolic health. It is also more effective than moderate-intensity training in reducing cardiovascular risk factors, inflammation, and insulin resistance (Ramos et al., 2015). Although few studies have investigated the effect of HIIT in individuals with OSA, they have found that HIIT can improve OSA severity, sleep quality, and CRF (Karlsen et al., 2016; Lins-Filho et al., 2024; Lins-Filho et al., 2023).

While these benefits have been observed in individuals with either OSA or T2DM, their effects on vascular health in those with both conditions remain unclear. Given the elevated cardiovascular risk in this population, it is important to study whether HIIT can improve flow-mediated dilation (FMD) and carotid intima-media thickness (CIMT), which are early markers of vascular function and structure. Assessing these outcomes is essential for understanding the potential vascular benefits of HIIT in individuals with coexisting OSA and T2DM. To address this gap, this pilot study examined the effects of HIIT on FMD and CIMT in adults with obesity, OSA, and T2DM, compared to CPAP and usual care. Secondary outcomes included changes in sleep indices, blood biomarkers, and anthropometric measures. We hypothesized a significant time × group interaction for vascular, sleep, and metabolic outcomes, indicating differential changes across the HIIT, CPAP, and usual care (control) groups.

## 2 Methods

### 2.1 Study population

This study recruited adults with obesity, OSA, and T2DM from the Snoring Clinic at Maharaj Nakorn Chiang Mai Hospital, Thailand, as well as through social media platforms, flyers, and word of mouth between July 2023 and April 2024. Potential participants were initially screened via telephone or in-person interview using a pre-screening checklist to confirm basic eligibility (e.g., age, body mass index (BMI), OSA/T2DM diagnosis). Participants were eligible if they met all of the following criteria: (1) classified as obese (BMI ≥ 30 kg/m^2^), according to the World Health Organization, (2) diagnosed with moderate-to-severe OSA (apnea-hypopnea index; AHI ≥ 15 events/h); (3) diagnosed with T2DM based on the American Diabetes Association criteria (fasting blood glucose (FBG) ≥ 126 mg/dL and/or Glycated hemoglobin (HbA1c) ≥ 6.5 %); (4) no comorbidities affecting respiratory or vascular outcomes (e.g., cardiovascular disease, liver disease, renal disease, pulmonary disease, or peripheral arterial disease, stroke, or transient ischemic attack); (5) no prior home CPAP use; (6) no diabetic nephropathy, retinopathy, or severe neuropathy; and (7) no participation in exercise programs or use of nutritional supplements in the past three months. Exclusion criteria included: (1) smoking within the past six months; (2) use of medications beyond usual care that could affect vascular function or structure (e.g., sedatives, hypnotics, corticosteroids); (3) uncontrolled hypertension (systolic ≥160 mmHg or diastolic ≥100 mmHg, or ≥ 140/90 mmHg while on antihypertensive medication); (4) significant weight change (≥ five kg within the past three months); and (5) any contraindication to exercise. Written informed consent was obtained from all participants. The study was approved by the Ethics Committee of the Faculty of Medicine, Chiang Mai University (Approval No. NONE-2565-0056) and conducted in accordance with the ethical principles of the Declaration of Helsinki. The trial was also registered in the Thai Clinical Trials Registry (TCTR20230821002).

### 2.2 Study design

This was a pilot, assessor-blinded, quasi-experimental study, matched participants by age range (20–29, 30–49, and 50–59 years) to reduce baseline age-related differences in vascular outcomes. Participants were sequentially assigned to HIIT, CPAP, or a control group by enrollment order within each age group. All groups received usual care, including lifestyle modification advice and medications, while maintaining their usual diet and physical activity. The primary outcomes were vascular health (FMD and CIMT), while secondary outcomes included sleep indices, blood biomarkers, blood pressure, and body composition.

### 2.3 Study procedure

Participants completed the Physical Activity Readiness Questionnaire and underwent safety screening by a qualified exercise physiologist. During the first visit in the hospital, overnight polysomnography (PSG) was conducted to assess OSA before starting any intervention. In the second visit, after an eight-hour fast, participants underwent blood sampling, body composition assessment, blood pressure measurement, and vascular health evaluation. Dietary intake and physical activity levels were tracked using the Global Physical Activity Questionnaire and dietary logs (two weekdays and one weekend day). Caloric intake was analyzed using the Thai Nutritional Surveys program. All outcomes were measured at baseline and after 12 weeks.

### 2.4 Measurements

#### 2.4.1 Primary outcomes

##### 2.4.1.1 Vascular function and structure

FMD and CIMT were measured by a trained technician. Participants refrained from strenuous exercise, high-fat foods, caffeine, and vitamin C for 24 h before testing. After resting supine for 15 min, the right brachial artery was scanned using pulsed wave Doppler ultrasound (Xario100, Toshiba, Japan) during a five-minute cuff occlusion at 50 mmHg above systolic blood pressure. Vascular diameter images were captured at baseline (FMDbase, mm) and 30-s intervals for two minutes after cuff release to assess peak dilation (FMDpeak, mm). FMD (%) was computed using the formula: ([FMDpeak–FMDbase]/FMDbase) × 100 (Thijssen et al., 2019). Then, a longitudinal ultrasound scan of the common carotid artery was conducted using an 11 MHz linear array transducer. CIMT was measured at one cm proximal to the carotid bulb between the lumen–intima and media–adventitia interfaces, averaging three measurements from both carotid arteries (Baumgartner et al., 2021). The intra-rater reliability for FMD and CIMT was 0.90 and 0.86, respectively.

#### 2.4.2 Secondary outcomes

##### 2.4.2.1 Sleep indices

Overnight PSG (SomnoLab2, V2.19, WM95410, Hamburg, Germany) was conducted by an experienced sleep technician following the American Academy of Sleep Medicine guidelines (Kapur et al., 2017). The procedure assessed sleep indices, including the AHI, oxygen desaturation index (ODI), mean oxygen saturation (SpO₂), and SpO_2_ nadir, which represents the lowest SpO₂ level during sleep.

##### 2.4.2.2 Blood biomarkers

Whole-blood samples (nine mL) were collected from the antecubital vein in the morning. For each patient, three plastic VACUETTE® blood collection tubes, each containing two to three mL of blood, were obtained: one tube with sodium fluoride for FBG measurement; one with dipotassium ethylenediaminetetraacetic acid for HbA1c measurement; and one without anticoagulants to determine lipid profiles: total cholesterol, high-density lipoprotein (HDL), low-density lipoprotein (LDL), and triglycerides. All tests were analyzed with an ARCHITECT ci8200 immunochemistry analyzer (Abbott Laboratories).

##### 2.4.2.3 Body composition and blood pressure

Body composition was assessed using a bioelectrical impedance analyzer (Tanita BC-418, Tokyo, Japan), measuring percent body fat (PBF), fat mass, and fat-free mass (FFM). BMI was calculated using weight (kg)]/[height (m^2^). Systolic blood pressure (SBP) and diastolic blood pressure (DBP) were measured in the dominant arm using a validated automatic monitor (HEM-7201, Omron Healthcare, Kyoto, Japan) after at least five minutes of seated rest, following American Heart Association guidelines. Three readings were taken one to two minutes apart, and the average was recorded.

### 2.5 Interventions

#### 2.5.1 HIIT protocol

Participants received education on proper clothing, sleep, hydration, carbohydrate intake, and dysglycemia prevention. The estimated maximal heart rate (HRmax) was calculated using the formula: [208 – (0.7 × age)] (Franckowiak et al., 2011), with adjustments for beta-blocker use incorporating ratings perceived exertion (RPE). Before starting the 12-week HIIT program, participants completed a two- to three-week familiarization phase, exercising at 65–80 % of HRmax during four-minute high-intensity intervals and 40–60 % of HRmax during three-minute active recovery periods. Workload (treadmill speed or incline) was gradually adjusted (≤ 5 % per week) to help participants safely reach and sustain 80–85 % of HRmax during intervals. Once achieved, they transitioned to the main program.

The 12-week HIIT protocol followed a fixed structure, consisting of four 4-min intervals of treadmill walking or running (Turbo 3.0, USA) at 85 %–95 % of HRmax, interspersed with three-minute active recovery periods at 50 %–60 % of HRmax, based on Silva et al. (Silva et al., 2019). Each session included a five-minute warm-up at 50 %–60 % HRmax and ended with a cool-down at 40–50 % HRmax. Heart rate was continuously monitored every minute using heart rate sensors (Polar H10, Finland), and blood pressure was measured during the fourth minute of each intense period (Accoson, England). RPE scale (6–20) was recorded during the final minute of each intense and recovery period. Training was conducted three times per week on non-consecutive days using a fixed protocol. Although treadmill speed and incline were not systematically recorded, progressive overload was inherently achieved by maintaining the prescribed target heart rate zones (percentage of HRmax). As participants' cardiovascular fitness improved, a higher workload (e.g., increased speed or incline) was required to sustain the same heart rate, thereby inducing a natural progression in training intensity across the 12-week intervention.

Sessions were supervised by a physiotherapist following clinical HIIT guidelines (Taylor et al., 2019). Exercise was terminated for abnormal symptoms, including angina, a ≥ 10 mmHg drop in SBP, SBP > 250 mmHg, DBP > 115 mmHg, shortness of breath, or other serious adverse events.

#### 2.5.2 CPAP group

Participants in the CPAP group used CPAP for 12 weeks, with pressure levels individually adjusted by a physician. Participants were instructed to use CPAP for ≥ four hours/night on 80 % of nights, targeting 80 % adherence. CPAP usage was recorded in a logbook and verified by CPAP machines.

#### 2.5.3 Control group

The control group received usual care and maintained their current lifestyle.

### 2.6 Statistical analysis

Descriptive statistics summarized participant characteristics, adherence rates, and adverse events. The Shapiro–Wilk test was used to assess data distribution. The multiple imputation procedure was used to handle the missing data. A 2 × 3 mixed model analysis of variance (ANOVA) was used to assess treatment effects, with time (baseline and post-intervention) as the within-subject factor and group (CPAP, HIIT, and CON) as the between-subject factor. Interaction effects between time and group were evaluated. Bonferroni post-hoc tests were applied for multiple comparisons when significant main effects were observed. Effect sizes were calculated using partial eta-square (ηp^2^). ηp^2^ was interpreted as follows: 0.01 = small, 0.06 = medium, and 0.14 = large effect. Analyses were conducted using SPSS 30.0 (SPSS Inc., Chicago, IL, USA), and statistical significance was set at *p* ≤ 0.05.

## 3 Results

### 3.1 Participant characteristics

**Fig. 1:**
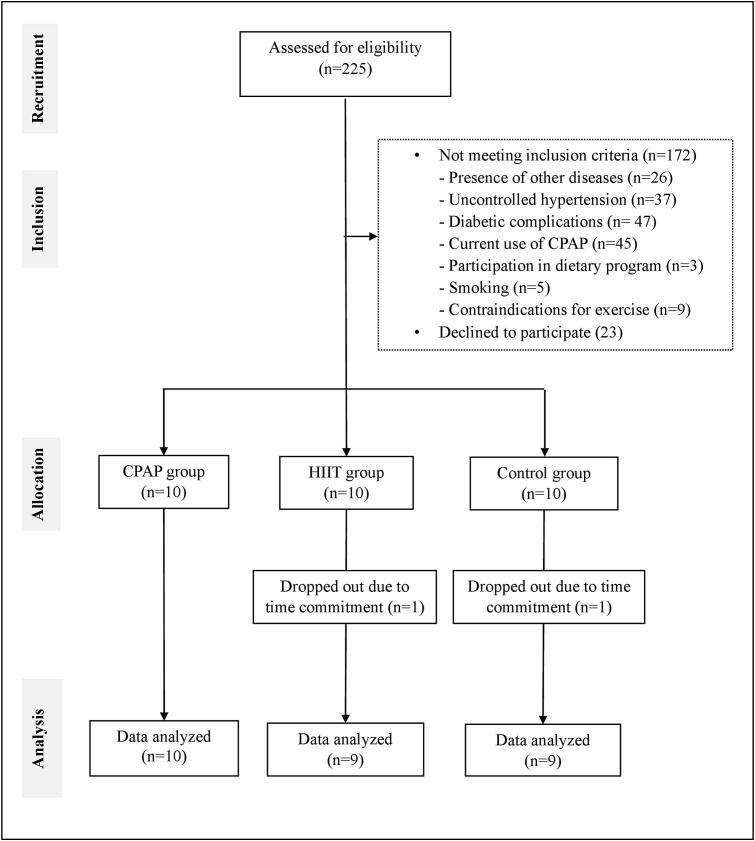
Flow diagram of participant selection among Thai adults with obesity, obstructive sleep apnea, and type 2 diabetes in Chiang Mai, Thailand, between July 2023 to April 2024.

Of 225 individuals screened with obesity, OSA, and T2DM, 30 met the eligibility criteria and were equally assigned to CPAP, HIIT, or control groups (10 per group). Two participants (one from the HIIT group and one from the control group) dropped out due to time constraints, resulting in a 6.67 % dropout rate. The participant flow (Fig. 1) and baseline characteristics (Table 1) are presented. As shown in Table 1, there were no significant differences in demographic or clinical variables between groups at baseline (all *p*-values>0.05). Mean adherence over 12 weeks was 97.75 % for HIIT and 95.89 % for CPAP. The mean duration of CPAP use was 6.2 h per night. During HIIT familiarization, all participants (100 %) in the HIIT group reported transient lower limb soreness, particularly in the thighs and calves, which subsided within seven to nine days. No significant musculoskeletal issues or adverse events occurred during the intervention period.

**Table 1:** Baseline characteristics and medication use among Thai adults with obesity, obstructive sleep apnea, and type 2 diabetes in Chiang Mai, Thailand, between July 2023 to April 2024.

Characteristics	Continuous positive airway pressure group (*n* = 10)	High-intensity interval training group (*n* = 9)	Control group (n = 9)	P-value
	n (%) / mean ± SD			
Age (years)	45.4 ± 10.0	37.2 ± 8.6	36.6 ± 10.2	0.10
Male	6 (60.0)	3 (33.3)	3 (33.3)	0.39
Diabetes duration (years)	4.1 ± 3.5	2.9 ± 2.0	2.2 ± 1.3	0.86
Apnea-hypopnea index (events/h)	17.2 ± 1.4	17.2 ± 1.5	16.7 ± 1.7	0.14
Body mass index (kg/m^2^)	34.5 ± 4.3	34.8 ± 1.5	37.9 ± 1.4	0.06
Physical activity (METs/week)	350.0 ± 365.2	426.7 ± 361.5	451.1 ± 371.1	0.65
Caloric intake (kcal/day)	1589.7 ± 205.7	1610.9 ± 284.5	1597.1 ± 128.5	0.46

Medications				
Anti-hyperglycemic	10 (100.0)	8 (88.9)	9 (100.0)	0.34
Anti-hypertensive	10 (100.0)	6 (66.7)	8 (88.9)	0.11
Lipid-lowering	6 (60.0)	7 (77.8)	8 (88.9)	0.34

P-values were derived from the analysis of variance.

### 3.2 Primary outcomes

The mixed-model repeated measures ANOVA revealed significant group-by-time interactions for FMD (*p* = 0.05) and CIMT (*p* = 0.02) (Table 2). Post-hoc analysis indicated a significant improvement in CIMT in the HIIT group compared to the control group (p = 0.05), while no significant differences were observed between groups for FMD (Table 3).

**Table 2:** Primary and secondary outcomes at baseline and 12 weeks post-intervention among Thai adults with obesity, obstructive sleep apnea, and type 2 diabetes in Chiang Mai, Thailand, between July 2023 to April 2024.

Variables		Continuous positive airway pressure group (n = 10)	High-intensity interval training group (n = 9)	Control group (n = 9)	Time P-value	Group P-value	Time x Group P-value	η_p_^2^
			mean ± SD					
Primary Outcomes: Vascular health								
Flow-mediated dilation (%)	Before	6.5 ± 1.7	6.3 ± 2.1	6.9 ± 1.9	0.03	0.56	0.05	0.21
	After	7.5 ± 3.8	9.8 ± 2.5	7.6 ± 3.3				
Carotid-intima media thickness (mm)	Before	0.6 ± 0.1	0.6 ± 0.1	0.6 ± 0.1	0.01	0.37	0.02	0.27
	After	0.6 ± 0.1	0.5 ± 0.1	0.6 ± 0.1				

Secondary Outcomes: Sleep indices								
Apnea-hypopnea index (events/h)	Before	17.2 ± 1.4	17.2 ± 1.5	16.7 ± 1.7	< 0.01	0.01	< 0.01	0.51
	After	12.1 ± 3.8	10.2 ± 3.6	17.00 ± 2.0				
Oxygen desaturation index (events/h)	Before	20.8 ± 12.3	13.6 ± 5.6	21.6 ± 12.5	0.75	0.11	0.08	0.19
	After	17.5 ± 10.0	11.0 ± 8.0	25.0 ± 14.5				
Mean oxygen saturation (%)	Before	92.8 ± 2.3	94.2 ± 1.5	93.5 ± 2.5	0.13	0.47	< 0.01	0.48
	After	95.4 ± 1.2	93.8 ± 1.4	92.9 ± 1.9				
Oxygen saturation nadir (%)	Before	76.3 ± 3.9	81.1 ± 3.9	79.0 ± 4.8	0.57	0.22	< 0.01	0.61
	After	84.9 ± 3.6	78.4 ± 7.2	75.0 ± 6.9				

Secondary Outcomes: Blood Biomarkers								
Total cholesterol (mmol/L)	Before	4.7 ± 1.0	4.2 ± 0.8	4.2 ± 0.8	0.21	0.35	0.84	0.01
	After	4.4 ± 0.9	3.9 ± 0.9	4.1 ± 1.2				
Triglyceride (mmol/L)	Before	1.5 ± 0.5	1.8 ± 0.8	1.9 ± 0.6	0.10	0.45	0.25	0.11
	After	1.5 ± 0.4	1.4 ± 0.4	1.7 ± 0.7				
High-density lipoprotein cholesterol (mmol/L)	Before	1.2 ± 0.2	1.2 ± 0.4	1.1 ± 0.3	0.28	0.33	0.26	0.10
	After	1.4 ± 0.5	1.2 ± 0.4	1.1 ± 0.3				
Low-density lipoprotein cholesterol (mmol/L)	Before	2.7 ± 0.8	2.6 ± 1.0	2.1 ± 0.5	0.09	0.56	0.78	0.17
	After	2.3 ± 0.9	2.7 ± 1.1	2.2 ± 0.9				
Fasting blood sugar (mmol/L)	Before	6.5 ± 1.0	6.6 ± 0.7	6.3 ± 1.1	0.89	0.55	0.58	0.04
	After	6.7 ± 1.7	6.5 ± 1.7	6.0 ± 0.6				
Glycated hemoglobin (%)	Before	7.1 ± 2.0	8.1 ± 2.6	6.7 ± 1.2	0.03	0.20	0.43	0.07
	After	6.8 ± 1.5	6.9 ± 1.2	5.9 ± 0.9				

Secondary Outcomes: Blood pressure and Body composition								
Systolic blood pressure (mmHg)	Before	137.2 ± 9.5	137.9 ± 11.0	141.1 ± 4.4	0.01	0.21	0.34	0.09
	After	133.6 ± 15.0	124.1 ± 3.8	134.4 ± 14.7				
Diastolic blood pressure (mmHg)	Before	89.9 ± 8.4	90.2 ± 2.2	93.8 ± 6.1	0.01	0.31	0.65	0.03
	After	87.0 ± 4.4	83.8 ± 8.5	89.3 ± 11.8				
Body mass index (kg/m^2^)	Before	34.5 ± 4.3	34.8 ± 1.5	37.6 ± 1.3	0.58	0.01	0.01	0.29
	After	33.8 ± 5.1	33.6 ± 1.2	39.0 ± 2.4				
Percent body fat (%)	Before	42.8 ± 6.7	42.5 ± 8.6	45.8 ± 8.4	<0.01	0.05	0.12	0.03
	After	41.0 ± 7.5	41.2 ± 8.2	44.7 ± 7.9				
Fat mass (kg)	Before	40.2 ± 6.9	44.8 ± 7.2	47.6 ± 7.3	0.05	0.06	0.15	0.14
	After	38.3 ± 8.4	38.3 ± 11.4	47.7 ± 7.3				
Fat-free mass (kg)	Before	54.2 ± 13.6	52.0 ± 9.7	46.8 ± 2.4	0.53	0.43	0.11	0.16
	After	51.5 ± 12.2	52.4 ± 10.1	48.3 ± 10.1				

P-values were derived from a 2 × 3 repeated measures analysis of variance. Effect sizes were calculated using partial eta-squared (η_p_^2^), interpreted as small (0.01), medium (0.06), and large (0.14).

**Table 3:** Between-group post-hoc comparisons of primary and secondary outcomes in Thai adults with obesity, obstructive sleep apnea, and type 2 diabetes in Chiang Mai, Thailand, between July 2023 to April 2024.

Variables	Continuous positive airway pressure group versus High-intensity interval training group		Continuous positive airway pressure group versus Control group		High-intensity interval training group versus Control group	
	Mean Differences (95 % confidence intervals)	η_p_^2^	Mean Differences (95 % confidence intervals)	η_p_^2^	Mean Differences (95 % confidence intervals)	η_p_^2^
Primary outcomes						
Flow-mediated dilation (%)	−2.33 (−6.20, 1.54)	0.18	−0.11 (−3.98, 3.77)	0.00	2.22 (−1.76, 6.19)	0.17
Carotid-intima media thickness (mm)	0.08 (−0.01, 0.17)	0.23	−0.01 (−0.10, 0.08)	0.00	−0.09 (−0.18, 0.00)	0.26

Secondary outcomes						
Apnea-hypopnea index (events/h)	1.89 (−1.99, 5.78)	0.13	−4.91 (−8.80, −1.02)	0.50	−6.80 (−10.79, −2.81)	0.66
Mean oxygen saturation (%)	1.62 (−0.18, 3.42)	0.17	2.50 (0.70, 4.30)	0.33	0.88 (−0.97, 2.72)	0.06
Oxygen saturation nadir (%)	6.46 (−0.64, 13.55)	0.30	9.90 (2.81, 16.99)	0.50	3.44 (−3.83, 10.72)	0.11
Body mass index (kg/m^2^)	0.18 (−3.85,4.21)	< 0.01	−5.18 (−9.21, −1.15)	0.43	−5.18 (−9.50, −1.21)	0.45

The estimated means were derived from a 2 × 3 repeated measures analysis of variance. Effect sizes were calculated using partial eta-squared (η_p_^2^), interpreted as small (0.01), medium (0.06), and large (0.14).

Within-group analysis showed FMD and CIMT improved significantly from baseline in the HIIT group (*p* < 0.01), whereas there were no significant changes in the CPAP or control groups (*p* > 0.05) (Table 4).

**Table 4:** Within-group post- hoc comparisons of primary and secondary outcomes in Thai adults with obesity, obstructive sleep apnea, and type 2 diabetes in Chiang Mai, Thailand, between July 2023 to April 2024.

Variables		Continuous positive airway pressure group		High-intensity interval training group		Control group	
		Mean Differences (95 % confidence intervals)	η_p_^2^	Mean Differences (95 % confidence intervals)	η_p_^2^	Mean Differences (95 % confidence intervals)	η_p_^2^
Primary outcomes							
Flow-mediated dilation (%)	Before-After	0.91 (−1.19, 3.02)	0.31	3.52 (1.31, 5.74)	0.30	−0.33 (−2.55, 1.88)	0.00
Carotid-intima media thickness (mm)	Before-After	−0.02 (−0.07, 0.03)	0.02	−0.10 (−0.15, −0.05)	0.39	0.00 (−0.05, 0.05)	0.00

Secondary outcomes							
Apnea-hypopnea index (events/h)	Before-After	−5.11(−7.18, −3.04)	0.51	−7.05 (−9.23, −4.87)	0.64	0.33 (−1.85, 2.50)	0.00
Mean oxygen saturation (%)	Before-After	2.61 (1.52, 3.70)	0.49	0.49 (−0.66, 1.63)	0.03	−0.64 (−1.79, 0.50)	0.05
Oxygen saturation nadir (%)	Before-After	8.60 (5.37, 11.83)	0.55	−2.67 (−6.10, 0.74)	0.09	−4.00 (−7.40, −0.58)	0.19
Body mass index (kg/m^2^)	Before-After	−0.70 (−1.88, 0.48)	0.06	−1.23 (−2.47, 0.02)	0.14	1.33 (0.09, 2.58)	0.16

The estimated means were derived from a 2 × 3 repeated measures analysis of variance. Effect sizes were calculated using partial eta-squared (η_p_^2^), interpreted as small (0.01), medium (0.06), and large (0.14).

### 3.3 Secondary outcomes

The mixed-model repeated measures ANOVA (Table 2) revealed significant time-by-group interactions for AHI, mean SpO₂, SpO₂ nadir, and BMI (*p* ≤ 0.01). There were no significant interactions for the remaining secondary outcomes, including ODI, total cholesterol, triglycerides, HDL, LDL, FBG, HbA1c, SBP, DBP, PBF, fat mass, and FFM (p > 0.05). Significant time effects were observed for HbA1c, SBP, DBP, and PBF (*p* < 0.05), with all groups showing reductions in these variables after 12 weeks. No significant time or group effects were observed in the remaining variables. Post-hoc analysis demonstrated significant reductions in AHI and BMI in both the CPAP and HIIT groups compared with the control group (p ≤ 0.01). The CPAP group exhibited significant increases in mean SpO₂ and SpO₂ nadir (p ≤ 0.01) (Table 3).

Within-group analysis showed significant reductions in AHI in both the CPAP and HIIT groups after 12 weeks (p < 0.01). The CPAP group exhibited significant increases in mean SpO₂ and SpO₂ nadir (p < 0.01). The HIIT group showed a significant decrease in BMI compared to baseline (*p* = 0.05), while the control group demonstrated a significant increase (*p* = 0.04) (Table 4).

## 4 Discussion

This pilot study demonstrated that the HIIT group exhibited a preliminary improvement in CIMT compared to the control group, while no significant CIMT change was observed in the CPAP group. Both FMD and CIMT improved from baseline in the HIIT group only. Both HIIT and CPAP interventions significantly reduced AHI and BMI compared to the control group. Notably, CPAP, but not HIIT, significantly increased mean SpO₂ and SpO₂ nadir compared to the control group. These findings suggest HIIT may offer vascular benefits and both interventions may improve OSA severity and body composition. Notably, only CPAP improved oxygenation during sleep. No serious adverse events occurred, and adherence to HIIT was high (97.75 %), suggesting that HIIT was safe and feasible for individuals with obesity, OSA, and T2DM.

A greater reduction in CIMT following HIIT may suggest a structural vascular benefit. Although the magnitude of change varied across studies, our findings align with prior research showing HIIT-induced reductions in arterial thickness among individuals with T2DM and other at-risk populations (Francois et al., 2018; Ghardashi-Afousi et al., 2020). These structural adaptations may reflect early signs of cardiovascular benefits. In contrast, CIMT did not improve in the CPAP group, consistent with a previous study reporting no CIMT change after three months of CPAP use (Ng et al., 2017), although another study reported a reduction after six months (Amin, 2016) or in severe OSA cases (AHI ≥ 50 events/h) (Assallum et al., 2021). This may suggest that detecting structural improvements with CPAP requires a longer treatment duration or participants with more severe OSA.

For FMD, our findings did not align with previous studies reporting greater improvements following HIIT in patients with T2DM (Abdi et al., 2021; Mitranun et al., 2014). Meanwhile, the CPAP group showed no significant FMD improvement compared to the control group, consistent with prior studies using three months of CPAP in individuals with or without T2DM (Bakker et al., 2020; Comondore et al., 2009). However, the outcome differs from that of Kayastha et al. (Kayastha et al., 2021), who observed increased FMD after 12 weeks of CPAP in patients with OSA. Additionally, meta-analyses (Xu et al., 2015) have shown significant FMD improvements with CPAP over periods ranging from four weeks to six months. These mixed findings may be attributed to variations in baseline endothelial function, OSA severity, or adherence. A longer duration of CPAP use may be needed to elicit measurable vascular changes.

The HIIT group demonstrated significant improvements in both FMD and CIMT from baseline. Our findings showed a significant FMD increase of approximately 3.75 % from baseline, consistent with a meta-analysis reporting an average FMD improvement of 4.31 % with HIIT across various populations, including patients with heart failure, coronary artery disease, and T2DM (Ramos et al., 2015). The observed improvements in FMD and CIMT may be driven by HIIT-induced shear stress, which enhances nitric oxide production, improves endothelial function, and promotes arterial remodeling (Wisløff et al., 2007). The repeated high-intensity bouts may optimize blood flow dynamics, reduce oxidative stress, and improve arterial compliance, thereby enhancing vascular function and structure. However, these remain speculative and require confirmation in future studies.

Regarding sleep indices, both HIIT and CPAP significantly reduced AHI compared to baseline and control group, with no significant difference between the two interventions. These findings align with previous studies showing that exercise training, including HIIT, can reduce OSA severity (Karlsen et al., 2016; Lins-Filho et al., 2023), potentially through mechanisms such as decreased pharyngeal fat, improved venous blood flow, decreased upper airway edema, and enhanced airflow during sleep (Torres-Castro et al., 2021). Improved cardiorespiratory fitness from HIIT may support better breathing control and oxygenation. Similarly, the reduction in AHI observed in the CPAP group is consistent with earlier research (Wang et al., 2022; Zhang et al., 2016), supporting its effectiveness in alleviating sleep-disordered breathing. Notably, only CPAP improved mean SpO₂ and SpO₂ nadir compared to the control group, highlighting its role in maintaining upper airway patency and preventing nocturnal oxygen desaturation, whereas HIIT alone may not sufficiently prevent oxygen desaturation during sleep. Neither intervention significantly affected ODI, possibly due to limitations in sample size or sensitivity of this measure.

Reductions in BMI were observed in both intervention groups compared to the control group, while BMI increased slightly in the control group, suggesting that structured interventions may be more beneficial than lifestyle advice alone. Our findings are consistent with prior studies (Andreato et al., 2018; Wang et al., 2022). The decrease in the HIIT group may be attributed to increased energy expenditure and fat oxidation (Zhang et al., 2016), while the reduction in the CPAP group could relate to improvements in fluid balance and hormonal regulation (Chen et al., 2021). Changes in HbA1c, SBP, DBP, and PBF across all groups were likely influenced by ongoing pharmacologic treatment. Neither HIIT nor CPAP significantly improved FBS or lipid profiles, suggesting that metabolic changes may require longer interventions, greater exercise volume, or dietary modifications (Feng et al., 2024).

This pilot study demonstrated high adherence to HIIT with no significant adverse events. Although all participants initially reported muscle discomfort during the familiarization phase, symptoms resolved within one week. This suggests that HIIT was feasible and generally well-tolerated in individuals with obesity, OSA, and T2DM. Regarding vascular outcomes, HIIT was associated with large effect sizes for reductions in CIMT compared to the control group (ηp^2^ = 0.26) and from baseline (ηp^2^ = 0.39), as well as for improvements in FMD from baseline (ηp^2^ = 0.30). These preliminary findings suggest that FMD and CIMT may serve as useful surrogate markers for evaluating non-pharmacological interventions in this population. Based on our preliminary results, HIIT may offer potential benefits for vascular function and structure, and could be considered as a complementary approach to CPAP. Specifically, HIIT may be helpful for patients awaiting CPAP initiation or facing challenges with nightly adherence.

The study has several limitations. As this was a pilot study with a small sample size, the findings should be interpreted with caution and confirmed with larger, well-powered randomized controlled trials. The generalizability of the results may be limited by the quasi-experimental design and single-center setting. Additionally, dietary intake was assessed through self-reported food logs, which may have introduced reporting bias and influenced metabolic outcomes. The use of estimated HRmax instead of cardiopulmonary exercise testing (CPET) is another limitation, as it may have affected the accuracy of training intensity prescription and the assessment of training responses. Future studies should consider incorporating CPET to enhance training individualization and better assess physiological adaptations. Conducting HIIT in a controlled laboratory setting may also limit the applicability of the findings to real-world conditions. Future studies should evaluate the effects of HIIT, CPAP, and their combination on vascular and cardiometabolic health in this high-risk population. Additionally, longer follow-up periods are needed to assess the long-term benefits of these interventions.

## 5 Conclusion

This pilot study suggests that HIIT may improve vascular structure, as indicated by a significant reduction in CIMT compared to usual care, with within-group improvements in both CIMT and FMD. No significant vascular changes were observed with CPAP. Both HIIT and CPAP reduced AHI and BMI, while only CPAP improved oxygen saturation. These preliminary findings support the potential vascular benefits of HIIT in individuals with obesity, OSA, and T2DM. Further research with larger samples is warranted.

## CRediT authorship contribution statement

**Kanpiraya Nithitsutthibuta:** Writing – original draft, Methodology, Investigation, Formal analysis, Conceptualization. **Sureeporn Uthaikhup:** Writing – review & editing, Supervision, Methodology, Conceptualization. **Nuntigar Sonsuwan:** Writing – original draft, Supervision, Methodology, Investigation, Conceptualization. **Jaruta Kunritt:** Writing – review & editing, Methodology, Investigation. **Khajornsak Tragoolpua:** Writing – original draft, Methodology, Investigation. **Arisa Parameyong:** Writing – review & editing, Methodology, Investigation. **Pongson Yaicharoen:** Writing – review & editing, Methodology, Investigation. **Sainatee Pratanaphon:** Writing – review & editing, Supervision, Methodology, Funding acquisition, Conceptualization.

## Informed consent

Informed consent was obtained from all individual participants included in the study.

## Ethical approval

All procedures performed in studies involving human participants were in accordance with the ethical standards of the Research Ethics Committee, Faculty of Medicine, Chiang Mai University (30 June 2023; RAS:0056), and with the 1964 Helsinki Declaration and its later amendments or comparable ethical standards.

## Declaration of generative AI and AI-assisted technologies in the writing process

During the preparation of this work, the authors used ChatGPT Plus in order to improve the readability and language of the manuscript. After using this tool/service, the authors reviewed and edited the content as needed and take full responsibility for the content of the published article.

## Source of funding

This work was supported financially by the Faculty of Associated Medical Sciences (AMS Research Grant no.18, 2023), 10.13039/501100002842Chiang Mai University.

## Declaration of competing interest

The authors declare that they have no known competing financial interests or personal relationships that could have appeared to influence the work reported in this paper.

## Data Availability

Data will be made available on request.
